# Sequential Venous Percutaneous Transluminal Angioplasty and Balloon Dilatation of the Interatrial Septum during Percutaneous Edge-to-Edge Mitral Valve Repair

**DOI:** 10.1155/2017/3652413

**Published:** 2017-08-09

**Authors:** Rezo Jorbenadze, Johannes Patzelt, Meinrad Gawaz, Peter Seizer, Harald F. Langer

**Affiliations:** University Hospital, Department of Cardiology and Cardiovascular Medicine, Eberhard Karls University Tuebingen, Tuebingen, Germany

## Abstract

Percutaneous edge-to-edge mitral valve repair (PMVR) is widely used for selected, high-risk patients with severe mitral valve regurgitation (MR). This report describes a case of 81-year-old woman presenting with severe and highly symptomatic mitral valve regurgitation (MR) caused by a flail of the posterior mitral valve leaflet (PML). PMVR turned out to be challenging in this patient because of a stenosis and tortuosity of both iliac veins as well as sclerosis of the interatrial septum, precluding the vascular and left atrial access by standard methods, respectively. We managed to achieve atrial access by venous percutaneous transluminal angioplasty (PTA) and balloon dilatation of the interatrial septum. Subsequently, we could advance the MitraClip® system to the left atrium, and deployment of the clip in the central segment of the mitral valve leaflets (A2/P2) resulted in a significant reduction of MR.

## 1. Introduction

Percutaneous edge-to-edge mitral valve repair (PMVR) has proven to be beneficial for patients with severe mitral valve regurgitation (MR), who are not eligible for conventional mitral valve repair. PMVR, however, can turn out to be challenging. For instance,* anatomical variations of vascular access and sclerosis of the interatrial* septum in some patients may represent obstacles, which render the procedure more challenging.

## 2. Case Report

**Figure 1:**
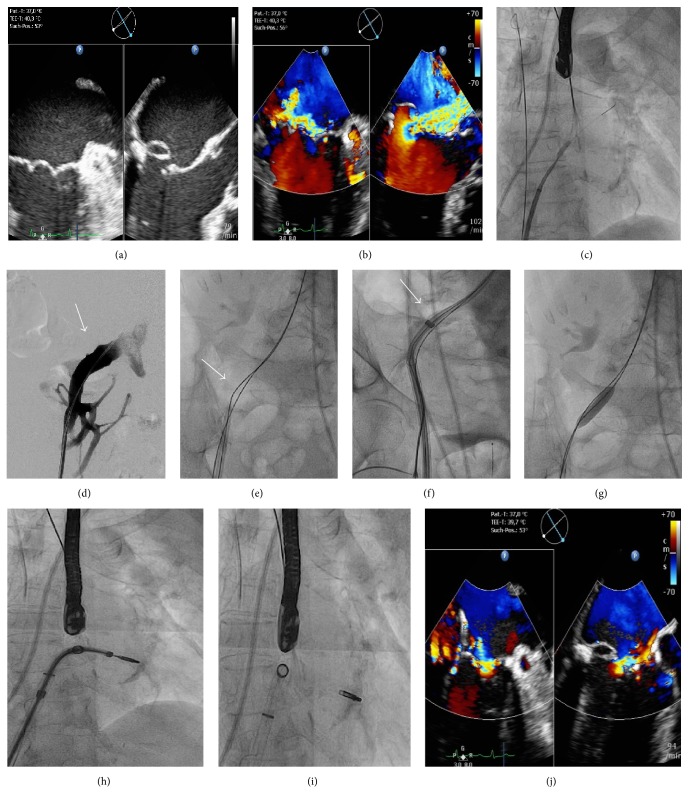
*Images of transesophageal echocardiography (TEE) and fluoroscopy during percutaneous edge-to-edge mitral valve repair (PMVR)*. (a) The biplane intercommissural and left ventricular outflow tract (LVOT) view in TEE showing a* severe* prolapse and flail of the posterior mitral leaflet (PML; P2 segment). (b) The biplane intercommissural and LVOT view in TEE with color Doppler demonstrating eccentric severe mitral regurgitation (MR). (c) Fluoroscopy showing balloon dilatation of the interatrial septum over a transseptal guidewire. (d) Digital substraction angiography (DSA): a mechanical obstruction in this area with a tortuous right common iliac vein precluded advancement of the MitraClip system (the depicted arrow may indicate a venous valve). (e) Fluoroscopy showing an unsuccessful attempt to dilate the right iliac vein with a dilator. (f) Unsuccessful attempt to advance the guide through the iliac vein (the arrow indicates an obstruction at the curve of the right common iliac vein). (g) Dilatation of the right iliac vein with increasing sizes of percutaneous transluminal angioplasty (PTA) balloons (up to 10 mm). (h) Successful positioning of the MitraClip delivery system and the clip within the left atrium. (i) Clip deployment. (j) Significant reduction of MR after clip deployment as demonstrated by intraprocedural TEE.

We report the case of* an* 81-year-old woman, who was admitted to our hospital with progressive dyspnea for further clinical evaluation. Transesophageal echocardiography (TEE) revealed severe MR with normal left ventricular systolic function. An eccentric jet was caused by a flail of the posterior leaflet (PML) in segment P2 (Figures 1(a) and 1(b)). Coronary angiography showed no significant coronary artery disease (CAD). Our interdisciplinary heart team recommended PMVR* because of the high risk of open surgery in this patient*.

After establishing vascular access through the right femoral vein, we* were not able to* advance the transseptal needle through the introduced Preface® transseptal access sheath (Biosense Webster, CA) across an obstruction at the curvature of the tortuous iliac veins. Instead, we had to use a thin transseptal guidewire (SafeSept®, Pressure Products, CA), which was advanced through the transseptal access sheath. This SafeSept wire* has* a very sharp tip and requires 77% less force to cross the interatrial septum. Using this approach, we then switched to a more rigid transseptal guiding sheath (LAMP, St. Jude Medical), and left atrial access could finally be achieved by dilatation* of the* interatrial septum with gradually increasing sizes of percutaneous transluminal coronary angioplasty (PTCA) balloons (up to 5 mm) over the SafeSept wire (Figure 1(c);* Supplemental Movie 1* in Supplementary Material available online at https://doi.org/10.1155/2017/3652413). Subsequently, we experienced another obstacle, as the dilator of the MitraClip steerable sheath system (Abbott Vascular) could not be advanced to the inferior vena cava over the placed ultrastiff wire (Amplatz, Cook Medical, Bloomington, IN), even when using a second superstiff buddy wire (Amplatz, Boston Scientific Corporation, San Jose, CA) (Figures 1(d), 1(e), and 1(f);* Supplemental Movie 2*). Furthermore, the left iliac vein had a similar obstruction precluding advancement of the MitraClip system. Thus, we carried out successive balloon dilatations of the right common iliac vein with gradually increasing balloon sizes up to 10 mm (Figure 1(g);* Supplemental Movie 3*). Deployment of the clip (Figures 1(h) and 1(i)) in the central segment of the mitral leaflets (A2/P2) resulted in a significant MR reduction (Figure 1(j)). We could discharge the patient from the hospital with a significantly improved 6-minute walk test few days after PMVR.

## 3. Discussion

PMVR presents a novel and innovative method for interventional mitral valve repair for patients with MR, who are not eligible for conventional heart surgery [1]. Less invasiveness and no need for extracorporeal circulation represent major advantages of PMVR over open heart surgery [2]. In some patients, however, anatomical circumstances may render PMVR difficult and in some cases even impossible. Besides difficult vascular access, existence of an atrial septal defect (ASD), a sclerotic interatrial septum, or a* relatively small* left atrium with additional presence of a prominent coumadin ridge,* which can make stirring towards the mitral valve plane difficult*, can complicate and prolong the procedure.* If a significant ASD is present, the position of the MitraClip steering guide may not be stable enough*. Crossing the iliac veins with the MitraClip guide catheter can represent a challenge during PMVR. Recently, a case of venous strangulation during PMVR was presented, which was overcome by implantation of a stent [3]. In our case, we were able to manage this vascular obstruction by percutaneous transluminal angioplasty (PTA) using PTA balloons of gradually increasing sizes. Multiple studies have shown that anticoagulation is needed after iliac venous stenting and sometimes even additional antiplatelet treatment is necessary for prevention of recurrent deep venous thrombosis [4]. In contrast, our approach has the advantage* where no prolonged antithrombotic therapy is required*. In addition to anatomical variation of the iliofemoral veins, thickening or lipomatous hypertrophy of* the* interatrial septum can represent a challenge for achieving* the* left atrial access. There are different methods for transseptal puncture,* for example, by using radiofrequency or excimer laser catheters*. Radiofrequency or laser energy* can be helpful* for transseptal puncture of a scarred, calcified, or patched atrial septum [5]. However, in our case we achieved the left atrial access via* a* SafeSept transseptal guidewire, a LAMP rigid transseptal guiding sheath, and gradual dilatation of* the* interatrial septum with PTCA balloons. We performed the transseptal puncture and the whole PMVR* procedure* under the guidance of TEE and fluoroscopy. However, intracardiac echocardiography (ICE) can also be used for monitoring of the* intervention* [6].

In conclusion, balloon angioplasty may be necessary for successful positioning of the MitraClip system and should be kept in mind as an option for establishing both vascular and left atrial access during PMVR.

## Supplementary Material

Supplemental Movie 1. Because of the sclerotic interatrial septum, we had to use a SafeSept® wire to cross the septum. Subsequently, balloon dilatation of the interatrial septum over the transseptal guidewire were carried out to be able to gain sufficient access to the left atrium.
Supplemental Movie 2. Because of a mechanical obstruction in the tortuous right common iliac vein advancing the MitraClip® steering guide or a commonly used dilator was not possible over the ultrastiff wire (Amplatz, Cook Medical, Bloomington, IN), even when using a second superstiff buddy wire.
Supplemental Movie 3. PTA using balloons with increasing diameter of up to 10 mm was carried out to overcome the mechanical obstruction of the iliac vein.

## References

[1] Feldman T., Foster E., Glower D. G. (2011). Percutaneous repair or surgery for mitral regurgitation. *The New England Journal of Medicine*.

[2] Beigel R., Wunderlich N. C., Kar S., Siegel R. J. (2014). The evolution of percutaneous mitral valve repair therapy: lessons learned and implications for patient selection. *Journal of the American College of Cardiology*.

[3] Al-Hijji M. A., Eleid M. F., Bjarnason H., Foley T. A., Reeder G. S., Rihal C. S. (2015). Venous strangulation as an unusual cause of mitraclip system delivery failure. *JACC Cardiovascular Interventions*.

[4] Eijgenraam P., ten Cate H., ten Cate-Hoek A. J. (2014). Venous stenting after deep venous thrombosis and antithrombotic therapy: a systematic review. *Reviews in Vascular Medicine*.

[5] Babaliaros V. C., Green J. T., Lerakis S., Lloyd M., Block P. C. (2008). Emerging applications for transseptal left heart catheterization: old techniques for new procedures. *Journal of the American College of Cardiology*.

[6] Henning A., Mueller I. I., Mueller K. (2014). Percutaneous edge-to-edge mitral valve repair escorted by left atrial intracardiac echocardiography (ICE). *Circulation*.

